# Comparison of Arterial Repair through the Suture, Suture with Fibrin or Cyanoacrylate Adhesive in *Ex-Vivo* Porcine Aortic Segment

**DOI:** 10.21470/1678-9741-2017-0107

**Published:** 2017

**Authors:** Marcus Vinicius H. de Carvalho, Evaldo Marchi, Edmir Américo Lourenço

**Affiliations:** 1 Department of Surgery, Faculdade de Medicina de Jundiaí, Jundiaí, SP, Brazil.

**Keywords:** Fibrin, Cyanoacrylates, Tissue Adhesives

## Abstract

**Introduction:**

Tissue adhesives can be used as adjacent to sutures to drop or avoid bleeding in cardiovascular operations.

**Objective:**

To verify the efficiency of fibrin and cyanoacrylate adhesive to seal arterial sutures and if the adhesives penetrate through suture line to the inner of arteries.

**Methods:**

20 abdominal aorta segments of pigs were divided into two groups according to the adhesive which would be used as adjacent to the suture. In every arterial segment an arteriotomy was done, followed by a conventional artery closure. Afterwards a colloidal fluid was injected inside the arterial segment with a simultaneous intravascular pressure monitoring up to a fluid leakage through the suture. This procedure was repeated after application of one of the adhesives on the suture in order to check if the bursting pressure increases. The inner aorta segments also were analyzed in order to check if there was intraluminal adhesive penetration.

**Results:**

In Suture 1 group, the mean arterial pressure sustained by the arterial suture reached 86±5.35 mmHg and after the fibrin adhesive application reached 104±11.96 (*P*<0.002). In the Suture 2 group, the mean arterial pressure sustained by the suture reached 83±2.67 mmHg and after the cyanoacrylate adhesive application reached 152±14.58 mmHg (*P*<0.002). Intraluminal adhesive penetration has not been noticed.

**Conclusion:**

There was a significant rise in the bursting pressure when tissue adhesives were used as adjacent to arterial suture, and this rise was higher if the cyanoacrylate adhesive was used. In addition, the adhesives do not penetrate through the suture line into the arteries.

**Table:** 

Abbreviations, acronyms & symbols	
PTFE	= Polytetrafluoroethylene
TA	= Tissue adhesives

## INTRODUCTION

Although arterial sutures are widely used in vascular surgeries, they present problems in some cases. These problems occur particularly in patients who are currently using anticoagulants, in those who present coagulopathy, and also in those cases in which vascular prostheses are necessary^[1]^. In the latter case, there may be bleeding problems in the needle hole. Certainly, tissue adhesives (TA) may be used as sealants to drop or avoid undesirable bleeding which can lead to morbidity and mortality rise. However, there is a range of products and, consequently, the need of an effective evaluation of a likely advantage from the TA use to avoid leakage in sutures is widely recognized^[2]^. Whenever TA is used as a complement to sutures, the TA should allow a bleeding-free artery closure, as well as through the needle holes. The present study aims to verify, on practical basis, the effectiveness of a biological origin adhesive, as well as a synthetic origin adhesive in sealing arterial sutures. This verification was done by checking the higher intravascular pressure level that can be supported before leakage occurs in the treated site, in order to compare the effectiveness of the two adhesives and to check if the adhesives penetrate in the inner surface of the vessel through the suture site.

## METHODS

The present study has been granted by the local Research Ethics Committee (CEUA-FMJ no. 230/2012). Segments of the abdominal infrarenal aorta were removed from slaughtered adult pigs (around 90 kg) in a slaughterhouse and promptly taken to the Surgical Technique Laboratory. These segments were prepared through adjacent tissue removal (keeping the adventitious layer tied to aorta) and through emergent branches tied with 4-0 cotton suture. One edge of the "Y" polypropylene tube (Compojet Biomédica, BA, Brazil) was firmly tied to the lumen of the aorta through 2-0 cotton suture. The aorta distal segment edge was fully occluded through a vascular clamp. After that, a saline solution at 0.9% NaCl was injected into the aorta segment in order to verify if there was any leakage through branches of the aorta and, in case of leakage, fix it (4-0 cotton suture). Whenever the aorta segments were leakage-free, a 100 mm longitudinal incision with no. 11 scalpel blade was done in each of them; this incision was sutured with a 7-0 polypropylene suture (Prolene^®^ Johnson & Johnson, Sao Jose dos Campos, Brazil) by a trained surgeon with binocular magnification x 2.5 loupes (Heine Optotechnik GmbH & Co KG^®^, Germany). Then, the aorta segments were considered prepared for the experiment.

### Bursting Pressure Test

**Fig. 1:**
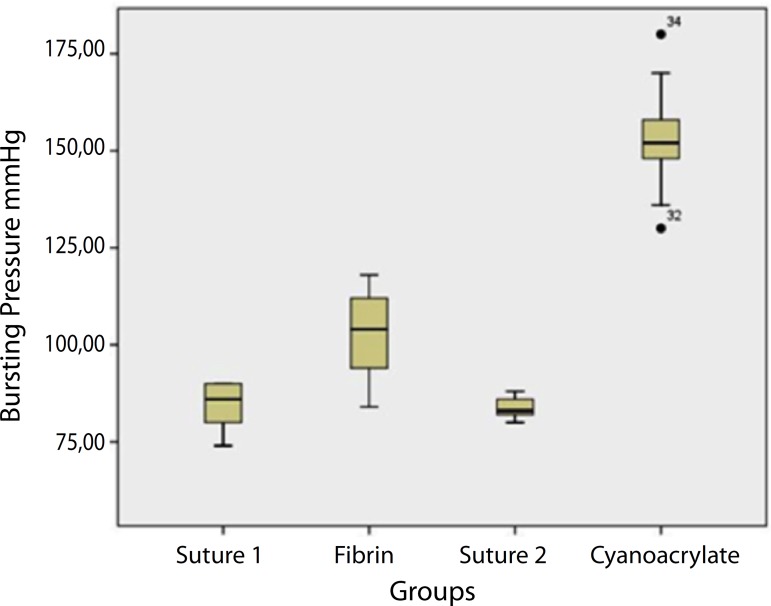
Results of bursting pressure test: pressure supported up to the moment of leakage occurrence, viewed by an observer with the aid of magnifying glass and proper illumination. Suture 1: aorta closure by suture; Fibrin: aorta closure by suture + fibrin adhesive; Suture 2: aorta closure by suture; Cyanoacrylate: aorta closure by suture + Cyanoacrylate adhesive.

In each prepared segment of the aorta, 6% of hidroethylamin solution (Voluven^®^ Fresenius Kabi, Barueri, Brazil) + 1 ml of 1% methyltioninium chloride (Azul de Metileno ADV^®^, Sao Paulo, Brazil) were injected through a "Y" catheter; the other end of the catheter was attached to a tensiometer (Missouri, table model, Mikatos Ind. Com., Sao Paulo, Brazil) to verify the intraluminal pressure. The objective of adding hidroethylamin is to make easier the visualization of fluid leakage. In ten aorta segments, the solution was injected slowly until the leakage occurs at the arterial suture site (Suture 1 group). The bursting pressure value was the mmHg pressure in which leakage occurred at the suture site. Then a fibrin patch was placed in the vessel at the suture site (Fibrin group) and, again, the bursting pressure was checked to see if there was any change in mmHg values. Also in another ten prepared segments of the aorta in which the arteriorrhaphy was already done, the solution was injected and the bursting pressure values were registered (Sutured 2 group). Thereafter, a film of cyanoacrylate adhesive was placed in the suture site and a new bursting pressure test was done (Cyanoacrylate group). The results are shown in Figure 1.

The occasional penetration of adhesive through the suture line into the artery by intervals between the stitches was evaluated by opening the arteries and viewing the inner layer by the surgeon through a 2.5 magnification loupe (Heine Optotechnik GmbH & Co KG^®^, Germany) and enough illumination.

### Statistical Analysis

For comparison among bursting pressure test values of Suture 1 group *versus* Suture 2 group and for Fibrin group *versus* Cyanoacrylate group, the Wilcoxon test for independent samples was used.

For comparison among bursting pressure test values of Fibrin group *versus* Suture 1 groups and among Suture 2 group *versus* Cyanoacrylate group, the signed-rank test was used.

## RESULTS

Bursting test pressure showed similar values between Suture 1 and Suture 2 groups (86±5.35 *versus* 83±2.67, *P*=0.3197).

In the Fibrin group, the median intraluminal pressure supported only by the suture was 86±5.35 mmHg and after application of the fibrin adhesive, this value reached 104±11.96 mmHg (*P*=0.002). In the Cyanoacrylate group, the median pressure supported only by the suture was 83±2.67 mmHg and after the application of this adhesive the median pressure reached 152±14.58 mmHg (*P*=0.0020).

The joining strength provided by the cyanoacrylate adhesive was significantly superior than that provided by the fibrin adhesive (152±14.58 *versus* 104±11.96, *P*<0.0002).

No trace of penetration of any adhesive within these vessels was found in any segment of the arterial aorta through intervals between stitches of the suture line.

## DISCUSSION

To date, there are no generally accepted rules or specifications for assessing TA performance and, due to this fact, many evaluation models have been proposed^[3]^. TA effectiveness would be better assessed by whether or not leakage of remaining fluids occurred. This endpoint, which can be easily and quickly checked, is, in most of circumstances, the clinically desirable endpoint^[3]^.

**Fig. 2:**
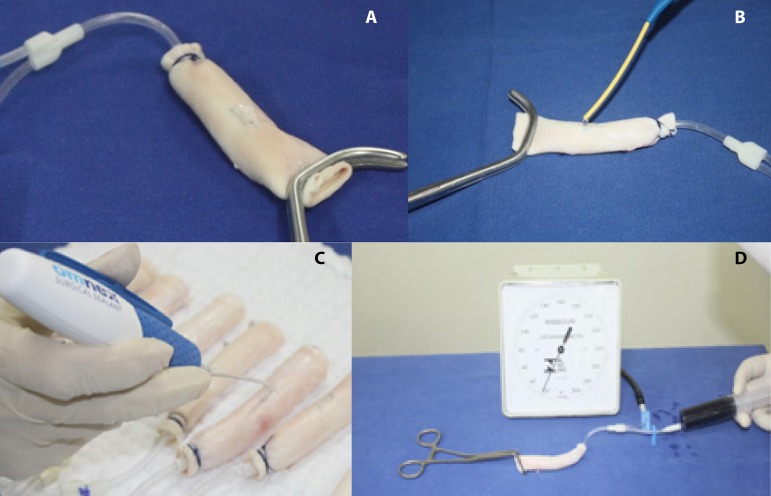
Details of all procedures. A) Aorta segment showing the 10 mm incision sutured. B) Application of fibrin adhesive over the sutured incision site. C) Application of cyanoacrylate adhesive over the sutured incision site. D) The set up to bursting pressure test.

For this study, an objective evaluation method was chosen, a method that is similar to that already utilized by Myers et al.^[4]^ (to evaluate the effectiveness of suture types) and by Flahiff et al.^[5]^ (to evaluate the effectiveness of the fibrin patch). This method consists of measuring the intraluminal pressure limit, in which the repair in the vessel area is sustained until leakage of intravascular fluid occurs through the suture line (Figure 2).

### Choice of Method for the Comparative Test

An *ex vivo* model can present a different performance when compared to an *ex vivo* model because there is no blood flow. A blood-flowing free tissue can compromise or raise the adhesive strength of some sealants, because some TAs depends on physical mechanisms to promote adhesiveness and other TA depend on enzymatic processes to work. Another critical parameter is the temperature. The impact of low temperatures could compromise the surgical adhesiveness of some sealants, presumably with a more emphatic impact in sealants which depend on human enzymatic activity^[6]^.The long-term response of the host tissue cannot be studied in the *ex vivo* model. Thus, these kinds of studies should use an animal model which can survive to evaluate the strength of cohesion over time, as well as the inflammatory markers. The selection of the animal model for leakage tests after the use of TA still remains a problem to be solved^[3]^. Chvapil et al.^[7]^ remind that, despite of the fact that several species of animals have been utilized to study bleeding, there are relevant hematological differences among animal species. Thus, the dog, for instance, presents higher platelet adhesion than the human. Bruck^[8]^ suggested that primate animals would be best suitable for testing because the blood and hemorrhage of these animals would best resemble the physiological characteristics of blood and bleeding in humans. However, these types of experiments would be impossible on the current days due to ethics evolution concerned with animal research and legislation. Therefore, the alternative for researchers is to perform tests using synthetic substances or biological tissue (*ex vivo*).

### Choice of Tissue Adhesives to Be Tested

The TA can be divided into two basic categories according to their origin, biological origin and synthetic origin. The main representative of the biological origin TA is the fibrin adhesive and the synthetic origin TA is the cyanoacrylate. The fibrin adhesive is made up of two basic components, the thrombin and the fibrinogen. The fibrinogen is the source of fibrin which represents the basic clotting element. Each of these ingredients is put into syringes apart from each other displayed side by side in a system that allows the mixing of the products only by the moment of application. The components interact along the application to form a stable fibrin clot. The relative rate of thrombin is that which will determine the rate of clot formation and the adhesion strength by the final phase of fibrin application^[9,10]^. As fibrin adhesives simulate the last clotting phase, they are autonomous and independent of body coagulation and, as a result, are also effective in patients who present coagulopathy as well in those who have been receiving anticoagulants^[11,12]^.

In contrast to synthetic adhesives, fibrin adhesives present the advantage of being biocompatible and biodegradable. The fibrin clot is reabsorbed within days or weeks as part of a normal wounds healing process. Macrophages and fibroblasts absorb the fibrin adhesive completely within about two weeks after application^[13,14]^. In this way, they are not associated with inflammation, foreign body reaction, tissue necrosis or extension of fibrosis^[15]^. However, in an extent review over fibrin sealants, Spotnitz^[16]^ demonstrated that, despite of the fact that the fibrin has been used for more than 25 years, it is still unclear whether it is actually effective in all types of suture.

Cyanoacrylates are liquid monomers which quickly polymerize when applied and then they quickly promote the adhesion of two altogether surfaces or a surface seal. Despite they were invented in 1942, the first formulations degraded into toxic products and only in 1998 new formulations became available, slowly degraded to allow medical use^[9]^. The advantages of the cyanoacrylates are fast application, the shortest repair period, and an effective fluid barrier. Cyanoacrylate has been effective in vascular reconstruction^[17,18]^, but there are still doubts about the possibility of the product particles penetrating into the vessel and then causing embolism^[19]^. The arterial wall tissues are more "elastic" than the vascular graft tissues, meaning that after the needle has crossed the arterial wall, a prompt tissue approach around the hole occurs, promoting fast occlusion. This does not occur in synthetic tissues grafts which present greater "memory" that maintain the needle hole open, which leads a trend in allowing the fluid adhesive to flow into the vessels in case of synthetic graft anastomosis^[17]^. It is also considered that the application of cyanoacrylate can cause an intense inflammatory reaction in the artery that reaches the internal wall of the vessels and this condition can lead to thrombus formation^[10]^.

### About the Results of the Present Study

The intraluminal pressure supported by suture was low in this experiment despite of the fact that the suturing was carefully done. It is certainly due to the use of a colloid solution rather than the use of blood with its coagulation factors. The cyanoacrylate sealant that has been used presents low viscosity, which permits a fast adhesion, but there is still a doubt about the limited elasticity. Cyanoacrylate showed a high strength cohesive power and a high bursting pressure in the present study, but it is also recognized for its stiffness. Thus, the low flexibility of cyanoacrylate can explain the failure in demonstrating its clinical advantages when compared to the lower cohesion strength TA^[20]^. The ideal TA should have an elastic flexibility, similar to the surrounding tissue to minimize the interface stress along the tissues drive^[20]^. This would be a disadvantage of cyanoacrylate. Kull et al.^[21]^ investigated the properties of cyanoacrylate in comparison to the fibrin adhesive and concluded that cyanoacrylate demonstrated a higher adhesion potential than fibrin in an *in vitro* porcine skin study. Others studies have also shown that cyanoacrylate adhesive has stronger power in bonding or sealing other tissues as lung^[22]^ and colon^[23]^ compared with other products.

Barbalinardo et al.^[24]^ have done the interposition of a 6 mm PTFE tube of iliac arteries utilizing sheeps and have found that the adjacent use of cyanoacrylate in the suture was effective as a synthetic graft anastomosis sealant. Saba et al.^[19]^ compared the graft interposition through continuous suture with 5-0 polypropylene and with an anastomosed suture-free graft with application of cyanoacrylate in the abdominal aorta of dogs. They came to the conclusion that cyanoacrylate can be a good alternative to do suture-free anastomosis. New studies will be necessary to confirm this alternative.

In the present study, the penetration of any adhesive through suture line into the artery segments did not occur. This fact confirms that the low viscosity of these adhesives makes it safe to use vascular surgery under this point of view. This study shows that cyanoacrylate adhesive provides higher bursting pressure and can sustain intravascular pressures up to 220 x 100 mmHg (mean arterial pressure 140 mmHg), even using a colloid solution instead of blood.

## CONCLUSION

There was a significant rise in the bursting pressure when tissue adhesives were used as adjacent to arterial suture, and this rise was considerably higher if the cyanoacrylate adhesive was used. The results show that the adhesives are effective sealants in arterial sutures in *ex vivo* models. In addition, the adhesives do not penetrate through suture line into the arteries.

**Table:** 

Authors' roles & responsibilities	
MVHC	Concept, design, acquisition, analysis and interpretation of data and critical review of the study; final approval of the version to be published
EM	Interpretation of data and critical review of the study; final approval of the version to be published
EAL	Interpretation of data and critical review of the study; final approval of the version to be published
